# The effects of mental health on recurrent falls among elderly adults, based on Korean Community Health Survey data

**DOI:** 10.4178/epih.e2020005

**Published:** 2020-02-02

**Authors:** Kyung Hee Jo, Jong Park, So Yeon Ryu

**Affiliations:** 1 Department of Public Health, Graduate School of Chosun University, Gwangju, Korea; 2 Department of Preventive Medicine, Chosun University Medical School, Gwangju, Korea

**Keywords:** Accidental falls, Mental health, Depression, Aged, Korea Community Health Survey

## Abstract

**OBJECTIVES:**

This study aimed to identify the effect of mental health on frequency of falls (single and recurrent falls) among elderly adults.

**METHODS:**

Data were drawn from the 2015 Korean Community Health Survey. A chi-square test was conducted to compare differences in fall frequency according to health-related behaviors, chronic diseases, and mental health. Subsequently, multinomial logistic regression analysis was used to identify the effects of mental health on single and recurrent falls based on variables found to be significant in the chi-square test.

**RESULTS:**

Recurrent falls were found to be more risky than single falls. Depression was significantly related to single falls (odds ratio [OR], 1.27; 95% confidence interval [CI], 1.12 to 1.44). Depression (OR, 1.56; 95% CI, 1.38 to 1.76), sleep disorder (5 hours or less: OR, 1.12; 95% CI, 1.02 to 1.23; more than 9 hours: OR, 1.24; 95% CI, 1.07 to 1.44, respectively), and subjective stress (OR, 2.30; 95% CI, 1.90 to 2.78) were significantly related to recurrent falls.

**CONCLUSIONS:**

The study’s findings suggest that specialized fall prevention programs are needed to address different types of falls in elderly adults. To prevent recurrent falls, systematic treatment strategies and rehabilitation training must improve physical function and mental health.

## INTRODUCTION

A fall refers to sustaining a physical injury from collapsing to the ground or tumbling down from a height, and can occur in people of all ages. In elderly adults, falling is the second most common cause of accidental death, following traffic accidents [1]. Approximately 30% of people aged 65 or over experience a fall, and 50% of those experience recurrent falls, making the factor a considerable threat to health in elderly populations [2].

In Korea, approximately 7.38 million people were 65 or older in 2018, constituting 14.3% of the country’s total population. Countries with an elderly population of 7% or more are generally classified as “aging societies”; countries like Korea, with an elderly population of 14% or more, are classified as “aged societies.” Furthermore, because Korea’s elderly population rate is continuing to increase, falls are a serious health issue [3].

Because bone density and muscle mass decrease with aging, elderly people’s bones can fracture with even a small shock. In the event of a fall, physical injuries are more likely to occur, including fractures, bruises, hematoma, edema, and other complications [4]. Mortality due to falls has steadily increased in those over 65, from 18.6 deaths per 100,000 population in 2015 to 20.3 in 2016 to 21.1 in 2017 [5]. The number of patients admitted to the hospital due to falls rose by 32% to approximately 124,000 between 2011 and 2015, with corresponding increases in socioeconomic costs [7]. Additionally, economic burdens are heightened when falls result in complications, which prolongs treatment and the related expenditures [6].

Major risk factors for falls, which interact in a complex fashion, include old age, female gender, living alone, drinking, chronic illness, previous experience of falls, low quality of life, depression, and dangerous environmental factors [8,9]. Recently, mental health has also been identified as a major risk factor. The risks of both falls in general and recurrent falls were particularly high in elderly adults with severe depression [10]. Depression in elderly adults decreases cognitive functioning and affects physical functioning, increasing the risk of falls [11,12].

Preventing single falls is difficult because they usually occur suddenly and by accident [13]. However, recurrent falls are common in older, feeble adults with numerous health complications or mental or physical problems [13]. Once elderly individuals experience one fall, anxiety and fear of falling again drives them to limit everyday activities, which reduces motor functioning and consequently increases their risk of a recurrent fall [14]. Because the mechanisms for single and recurrent falls are different, it is crucial to differentiate fall cases according to frequency and compare them accordingly [14].

Many studies have examined factors influencing falls in elderly adults. Most studies conducted in Korea have identified risk factors using a fall risk assessment model in small study samples [15-17], and studies on the relationship of mental health to falls have examined only the effect of depression [18,19]. Thus, the effects of mental health on falls have not been sufficiently investigated. The present study aimed to differentiate and examine the characteristics of individuals who experienced single and recurrent (two or more) falls using community-based data, and thereby to explore the effects of mental health on single and recurrent falls.

## MATERIALS AND METHODS

### Study materials and subjects

This study used raw data from the 2015 Korean Community Health Survey (KCHS) managed by the Korean Centers for Disease Control and Prevention. The KCHS survey population included adults aged 19 or older living in each of the country’s regions. After selecting sampling units using a probability proportional to size method, systematic sampling was used to select households from within each of the sampling units. Data from approximately 900 individuals were collected at 254 public health centers nationwide, and trained survey conductors visited subjects in the sampled households between August 31, 2015 and November 8, 2015. The survey included a total of 198 items across 19 domains and was administered via one-on-one interview using a laptop on which the survey program was installed [20].

Of the 228,558 total survey respondents in the 2015 KCHS, 63,929 were over age 65. The present study’s analysis included 63,899 respondents, excluding 30 due to incomplete responses to the items regarding experience of falls within the past year and number of accidental falls.

### Study variables

To identify factors influencing falls in elderly adults over 65, this study examined socio-demographic factors, health behaviors, physical health-related variables, and mental health-related variables.

#### Fall-related variables

Experience of falls was categorized as “none,” “once,” or “twice or more,” based on the number of falls experienced by respondents who responded yes to the question on whether they fell within the past year. Fear of falls was categorized as “not at all afraid,” “a little afraid,” or “very afraid,” based on the question, “In general, are you afraid of falling?” Treatment due to falls during the past year was categorized as “yes” or “no” based on the question, “Have you fallen in the past 1 year?” Being bedridden was also categorized as “yes” or “no” based on the question, “In the past 1 month, have you been bedridden for almost all day due to an illness or injury?”

#### Socio-demographic factors

Socio-demographic factors included gender, age, education level, marital status, living arrangement, monthly income, residence type, locality, and national basic livelihood recipient status. Gender was categorized as men or women. Education level was grouped into five categories according to the respondents’ final year of education and whether they graduated: no formal education, elementary school, middle school, high school, and college or higher. Marital status was categorized as either having a spouse if the respondent had a spouse, or having no spouse if the respondent was divorced, separated, widowed, or not married. Categories for living arrangement included living alone, living with spouse, and living with family or others. Monthly income was categorized as less than 1 million Korean won (KRW), 1-2 million KRW, 2-3 million KRW, or 3 million KRW or more. Residence type was classified as “house” or “apartment,” and locality was classified as “urban” if the respondent lived in a *dong* (neighborhood) in the administrative division or as “rural” if the respondent lived in an *eup* (town) or *myeon* (township). National basic livelihood recipient status was classified as “yes” or “no.”

#### Health behaviors

Smoking was classified as “yes” for respondents who answered that they smoked every day or occasionally, and as “no” for respondents who answered that they did not smoke, or smoked previously but not currently. Drinking was categorized as “no (less than once a month),” “low to moderate (1-4 times per month),” and “high risk (twice or more per week)”; specifically “high-risk” drinking was defined as drinking over seven glasses of soju for men and over five glasses for women twice per week or more. For physical activity, moderate intensity was coded “yes” if the respondent exercised for 20 minutes or more at least three days a week, and if the high intensity was coded “yes” if the respondent exercised for 30 minutes or more at least five days a week. Walking was coded “yes” for respondents who walked for 30 minutes or more at least five days a week. Subjective health was classified as “good,” “average,” or “poor” based on the question, “In general, how do you perceive your health?” Body mass index (BMI) was computed using respondents’ height and weight, and classified as “underweight (<18.5 kg/m^2^),” “healthy weight (18.5-24.9 kg/m^2^),” “obese (25.0-29.9 kg/m^2^),” and “severely obese (≥ 30.0 kg/m^2^).”

#### Physical health-related variables

Hypertension, diabetes, dyslipidemia, and arthritis were coded “yes” or “no” according to whether the respondent was currently receiving treatment for the corresponding condition. Number of chronic illnesses was coded as 0, 1, 2, or 3 based on the total number of conditions (hypertension, diabetes, and arthritis) the respondent was receiving treatment for.

#### Mental health-related variables

Subjective stress was categorized as “very much,” “much,” “a little,” and “rarely” based on the question, “In general, how stressed do you feel in everyday life?” Depression was coded as “yes” or “no” based on the question, “In the past 1 year, have you felt sadness or despair to the extent that you could not complete everyday activities for 2 consecutive weeks or more?” Daily sleep duration was categorized as “5 hours or less,” “6-8 hours,” and “more than 9 hours,” with 5 hours or less or more than 9 hours being defined as sleep disorder [21].

### Statistical analysis

All statistical analyses were performed using SPSS version 25.0 (IBM Corp., Armonk, NY, USA). Independent variables with the potential to influence falls were selected based on a review of the literature on falls. The statistical analysis accounted for the complex sample design taken into account, with individual weights computed and applied to the population. To examine the differences between the single and recurrent fall groups according to each of the variables, a chi-square test was performed. To examine between-group differences with respect to the relevant variables, complex-sample multinomial logistic regression analysis was conducted with fall frequency as the dependent variable and individuals over 65 as the study population to estimate odds ratios (ORs). Statistical significance was determined by a p-value of less than 0.05.

### Ethics statement

The raw data were requested from the KCHS homepage (http://chs.cdc.go.kr/), and were obtained with all private information remaining anonymous.

## RESULTS

### Subjects’ fall-related characteristics

Among the study’s subjects, 19.6% had experienced at least one fall within the past year and 80.4% had not. Over the past year, 11.6% had fallen once and 8.0% had fallen twice or more; 48.1% received treatment for a fall and 51.9% did not (Table 1). A total of 38.8% indicated they were “not afraid at all” of falling, 37.7% were “a little afraid,” and 23.5% were “very afraid.” Fall experiences, overall falls, and recurrent falls were highest among those with the greatest fear of falling. Of all subjects, 8.1% had been bedridden and 91.9% had not. Those who had been bedridden had higher rates of fall experiences and recurrent falls (Table 2).

### General characteristics and health behaviors according to fall frequency

Fall experiences and recurrent falls were higher in men than women. Mean age was 73.1 years in the group with no experience of falls, 73.9 in the single fall group, and 74.8 in the recurrent fall group, showing an increase in fall frequency with increasing age. Falls were more frequent in individuals without a spouse than in those with a spouse, and among those living alone compared to those living with a spouse. The rate of single falls was higher in the urban area, and the rate of recurrent falls was higher in the rural area. National basic livelihood recipients demonstrated higher rates of fall experience and recurrent falls. Fall experience and recurrent falls were higher in those who did not smoke or drink than in those who did smoke or drink. Respondents who engaged in walking or high/moderate intensity physical activity had lower rates of fall experience and recurrent falls. Respondents whose subjective health was poor had higher rates of fall experience and recurrent falls. Fall experience was higher in severely obese individuals, and recurrent falls were most common in both underweight and severely obese individuals. Between-group differences were statistically significant for all the measured variables (Table 3).

### Mental health and chronic illness according to fall frequency

Higher rates of single and recurrent falls were found in respondents who slept 5 hours or less or more than 9 hours a day; had higher subjective stress; had experienced depression; were currently receiving treatment for hypertension, diabetes, dyslipidemia, or arthritis; and had a higher number of chronic illnesses. Additionally, rates of single and recurrent falls increased with decrease in respondents’ subjective health status. All variables related to mental health and chronic illness were statistically significantly different between groups (Table 4).

### Effects of mental health on frequency of falls

Multinomial logistic regression analysis showed that in the single fall group, fall risk was higher in elderly adults with experience of depression (OR, 1.27; 95% confidence interval, 1.12 to 1.44). In the recurrent fall group, fall risk was higher in elderly adults with experience of depression (OR, 1.56; 95% CI, 1.38 to 1.76) and in those who slept for 5 hours or less or more than 9 hours (OR, 1.12; 95% CI, 1.02 to 1.23; OR, 1.24; 95% CI, 1.07 to 1.44, respectively). Additionally, risk of recurrent falls was greater in those with high subjective stress (OR, 2.30; 95% CI, 1.90 to 2.78) than in those with only a little subjective stress (OR, 1.21; 95% CI, 1.09 to 1.34) (Table 5).

## DISCUSSION

Health status influences the risk of falls in elderly adults, and this includes not only physical health but also mental health. In elderly adults with more vulnerable health status, single falls are more likely to recur and have a serious effect on health, potentially leading to death [13].

In Korea, approximately 19.6% of adults 65 or older experienced at least one fall within the past year. Of those, 59.2% experienced a single fall and 40.8% experienced recurrent falls (two or more). A study of 329 community-dwelling elderly adults in a rural area found that 73.3% experienced single falls and 21.7% experienced recurrent falls, respectively [22]. Another found that among 104 community-dwelling elderly adults who were registered at regional public health centers and welfare service centers, 55.7% experienced a single fall and 44.2% experienced recurrent falls [15]. However, these previous studies were conducted with non-representative samples, which could mean that inconsistencies in the subjects’ characteristics affected the studies’ findings. Thus, a precise understanding of falls in the Korean elderly population has thus far been difficult. To fill this gap, the present study used data from the 2015 KCHS to investigate the effects of mental health on falls in elderly adults, differentiating between single and recurrent fall cases.

The study found that risk of single falls increased with depression, and that risk of recurrent falls increased with depression, sleep disorder (sleeping 5 hours or less or more than 9 hours per day), and greater subjective stress. Thus, mental health was demonstrated to have a greater effect on recurrent falls among elderly adults. Additionally, people who experience recurrent falls tend to reduce their physical activity in everyday life due to fear of falling, which affects them emotionally and increases the likelihood of falling again [15].

Among people who fell once, fall risk significantly increased by 1.27 times in those who experienced depression. This is consistent with a study conducted outside Korea, which demonstrated that greater depression was associated with higher risk of falls in elderly adults [23]. A study conducted in Korea also found a higher risk of falls in respondents with depression [19]. Depression deteriorates mental functions in elderly adults, lowering their cognitive functioning and also affecting their physical functioning. These mechanisms are speculated to increase the risk of falls [11,12]. In those who experienced recurrent falls, depression increased the risk of falls by 1.56 times, a greater risk than in the single fall group. Fall recurrence has been reported to correlate highly with depression [21], with a 45% higher rate of fall recurrence in patients with depression [24]. Additionally, research has reported that a major factor in recurrent falls is that after an initial fall, subjects’ physical functioning and social activity decrease due to physical injury, consequently increasing depression [18].

Mental health is closely associated with sleep duration and quality. Conventionally, a sleep duration of 5 hours or less or more than 9 hours is defined as sleep disorder [21]. In this study, individuals with sleep disorder in the recurrent fall group tended to have increased fall risk. This risk was particularly increased for those who overslept (more than 9 hours). Some studies report that sleep quality may be much lower in oversleeping (more than 9 hours) than sleep deprivation (5 hours or less) [25]. Poorer sleep quality deteriorates physical health and affects mental and cognitive functioning, which can influence falls [26], and the risk of another fall increases in elderly individuals who have already fallen [27]. However, various aspects must be considered when assessing sleep, including amount of sleep, number of arousals, and sleep latency [28], so it is difficult to assess sleep based on duration alone. Research examining the effects of sleep on recurrent falls is also extremely scarce. Thus, future research on this topic is needed.

Subjective stress was also found to influence falls in the recurrent fall group, indicating that higher subjective stress increases the risk of fall recurrence. Whereas a single fall tends to occur due to a sudden accident, recurrent falls occur because fear of falling increases with recurrent fall experiences, adding to psychological stress [13]. These negative effects eventually impact individuals’ physical activity and functional recovery capacity [29]. Accordingly, mental health promotion programs to enhance self-efficacy and reduce stress should be provided for elderly individuals who experience recurrent falls, in parallel with training to regain physical functioning.

This cross-sectional study confirmed the effects of mental health on falls among elderly adults. Additionally, the results demonstrated that mental health had greater effects on recurrent falls. A variety of complex factors are speculated to affect elderly adults’ emotional states, for example increasing their sleep and subjecting them to stress and depression, but it was difficult to explain the temporal relationships among mental health variables. Additionally, the study’s results cannot describe the directionality of the relationship between recurrent falls and mental health. However, this study is significant in that it was based on a representative, community-based database and demonstrated the differing effects of mental health on risk of single and recurrent falls.

In conclusion, the risk of recurrent falls may increase in Korean elderly adults as mental health status grows more vulnerable through stress, depression, and sleep disorder. Whereas single falls tend to occur due to a sudden accident, recurrent falls primarily occur in high-risk groups. Therefore, it is possible to prevent recurrent falls. Accordingly, fall prevention programs should be developed to manage specific risk factors according to individuals’ fall frequency. For people experiencing recurrent falls in particular, systematic treatment strategies and rehabilitation training are necessary to improve both mental health and physical functioning.

## Figures and Tables

**Table 1.** Fall-related characteristics of adults aged 65 and older (n=63,899)

Variables	n	% (% standard error)
Fall experience		
Yes	12,659	19.6 (0.2)
No	51,240	80.4 (0.2)
No. of falls in the past year		
0	51,240	80.4 (0.2)
1	7,190	11.6 (0.2)
≥ 2	5,469	8.0 (0.1)
Treatment for a fall within the past year		
Yes	5,972	48.1 (0.6)
No	6,687	51.9 (0.6)

**Table 2.** Fall-related characteristics according to fall status in adults aged 65 and older

Variables	Total (%)	Fall status			p-value^1^
		No	Single	Recurrent	
Fear of falling					
Not at all afraid	38.8	90.4 (0.3)	6.8 (0.2)	2.8 (0.1)	<0.001
A little afraid	37.7	81.1 (0.4)	11.9 (0.3)	7.0 (0.2)	
Very afraid	23.5	62.8 (0.5)	19.0 (0.4)	18.1 (0.4)	
Bedridden in past month					
Yes	8.1	61.8 (0.9)	17.2 (0.7)	21.0 (0.8)	<0.001
No	91.9	82.1 (0.2)	11.1 (0.2)	6.8 (0.1)	

Values are presented as % (% standard error).

1 Chi-square test.

**Table 3.** Differences in general characteristic and health behaviors by fall status in adults aged 65 and older

Variables	Total (%)	Fall status			p-value^1^
		No	Single	Recurrent	
General information (n)	63,899	51,240	7,190	5,469	
Gender					<0.001
Men	43.2	76.3 (0.3)	14.0 (0.2)	9.7 (0.2)	
Women	56.8	85.9 (0.2)	8.4 (0.2)	5.7 (0.2)	
Age, mean±SE (yr)	73.91 ± 0.06	73.08 ± 0.04	73.85 ± 0.10	74.80 ± 0.12	<0.001
Education level					<0.001
No formal education	26.0	87.2 (0.7)	9.1 (0.6)	3.8 (0.4)	
Elementary school	31.1	84.8 (0.5)	9.3 (0.4)	5.9 (0.3)	
Middle school	15.4	83.2 (0.6)	11.2 (0.5)	5.6 (0.3)	
High school	19.4	84.8 (0.5)	9.3 (0.4)	5.9 (0.3)	
College or higher	8.1	87.2 (0.7)	9.1 (0.6)	3.8 (0.4)	
Marital status					<0.001
No spouse	35.8	75.4 (0.4)	14.0 (0.3)	10.6 (0.3)	
Spouse	64.2	83.2 (0.3)	10.3 (0.2)	6.5 (0.2)	
Living arrangement					<0.001
Alone	19.1	75.3 (0.5)	13.7 (0.4)	11.0 (0.3)	
With spouse	43.8	83.4 (0.3)	10.1 (0.2)	6.5 (0.2)	
With family or others	37.1	79.5 (0.4)	12.3 (0.3)	8.2 (0.3)	
Monthly income (10^4^ Korean won)					<0.001
<100	43.2	77.3 (0.3)	12.7 (0.3)	10.0 (0.2)	
100-200	22.4	82.5 (0.4)	10.9 (0.4)	6.5 (0.3)	
200-300	14.0	83.3 (0.6)	10.7 (0.5)	6.0 (0.4)	
≥300	20.4	82.7 (0.5)	10.7 (0.4)	6.5 (0.3)	
Residence type					<0.001
House	64.7	81.3 (0.4)	11.7 (0.3)	7.0 (0.3)	
Apartment	35.3	79.9 (0.2)	11.6 (0.2)	8.5 (0.2)	
Locality					<0.001
Urban	69.9	80.6 (0.3)	11.8 (0.2)	7.6 (0.2)	
Rural	30.1	80.1 (0.3)	11.0 (0.2)	8.9 (0.2)	
National basic livelihood recipient status					<0.001
Yes	6.3	70.9 (1.0)	15.1 (0.8)	14.0 (0.7)	
No	98.7	81.1 (0.2)	11.4 (0.2)	7.6 (0.1)	
Smoking					<0.001
Yes	9.3	84.6 (0.6)	9.4 (0.5)	5.9 (0.4)	
No	90.7	80.0 (0.2)	11.8 (0.2)	8.2 (0.2)	
Drinking					<0.001
None	65.9	78.8 (0.3)	12.4 (0.2)	8.9 (0.2)	
Low to moderate	23.2	83.3 (0.4)	10.2 (0.4)	6.5 (0.5)	
High-risk	10.9	85.0 (0.6)	9.6 (0.5)	5.4 (0.4)	
High intensity exercise					<0.001
Yes	9.8	83.6 (0.7)	9.9 (0.6)	6.5 (0.4)	
No	90.2	80.1 (0.2)	11.8 (0.2)	8.1 (0.2)	
Moderate intensity exercise					<0.001
Yes	19.0	83.5 (0.4)	10.1 (0.4)	6.4 (0.3)	
No	81.0	79.7 (0.2)	12.0 (0.2)	8.3 (0.2)	
Walking					<0.001
Yes	51.6	83.0 (0.3)	10.7 (0.3)	6.3 (0.2)	
No	48.4	77.7 (0.3)	12.6 (0.2)	9.7 (0.2)	
Subjective health status					<0.001
Good	21.6	88.7 (0.4)	8.1 (0.3)	3.3 (0.2)	
Average	36.5	83.5 (0.3)	11.2 (0.3)	5.3 (0.2)	
Poor	41.9	73.5 (0.4)	13.8 (0.3)	12.7 (0.3)	
Body mass index (kg/m^2^)					<0.001
Underweight (<18.5)	12.3	77.0 (0.6)	12.5 (0.4)	10.6 (0.4)	
Healthy weight (18.5-24.9)	64.6	81.4 (0.3)	11.4 (0.2)	7.2 (0.2)	
Obese (25.0-29.9)	21.3	80.3 (0.5)	11.3 (0.4)	8.5 (0.5)	
Severely obese (≥30.0)	1.8	74.2 (2.1)	15.2 (1.8)	10.6 (1.5)	

Values are presented as % (% standard error).

1 Chi-square test.

**Table 4.** Differences in mental health and chronic illness characteristics by fall status in adults aged 65 and older

Variables	Total (%)	Fall status			p-value^1^
		No	Single	Recurrent	
Sleep duration (hr)					<0.001
≤5	25.5	77.5 (0.4)	12.4 (0.4)	10.1 (0.3)	
6-8	68.7	81.8 (0.3)	11.2 (0.2)	7.0 (0.2)	
≥9	5.8	76.7 (0.9)	13.2 (0.7)	10.1 (0.6)	
Subjective stress level					<0.001
Rarely	35.1	83.3 (0.3)	10.9 (0.3)	5.7 (0.2)	
A little	44.7	81.6 (0.3)	11.5 (0.3)	6.9 (0.2)	
Much	17.3	73.5 (0.6)	13.1 (0.5)	13.4 (0.5)	
Very much	2.9	67.7 (1.4)	13.1 (1.0)	19.3 (1.2)	
Depression					<0.001
Yes	8.3	67.7 (0.9)	15.1 (0.7)	17.2 (0.8)	
No	91.7	81.3 (0.2)	11.3 (0.2)	7.1 (0.1)	
Hypertension					<0.001
Yes	51.0	79.3 (0.3)	12.0 (0.2)	8.7 (0.2)	
No	49.0	81.5 (0.3)	11.2 (0.3)	7.2 (0.2)	
Diabetes					<0.001
Yes	20.3	77.2 (0.5)	13.3 (0.4)	9.6 (0.4)	
No	79.7	81.3 (0.2)	11.2 (0.2)	7.6 (0.2)	
Hyperlipidemia					<0.001
Yes	18.4	77.7 (0.6)	13.0 (0.4)	9.2 (0.4)	
No	81.6	81.1 (0.2)	11.3 (0.2)	7.7 (0.2)	
Arthritis					<0.001
Yes	18.7	71.2 (0.5)	14.7 (0.4)	14.1 (0.4)	
No	81.3	82.6 (0.2)	10.9 (0.2)	6.6 (0.1)	
No. of chronic illnesses					<0.001
0	35.7	83.8 (0.3)	10.3 (0.3)	5.9 (0.2)	
1	41.5	80.6 (0.3)	11.5 (0.3)	7.9 (0.2)	
2	19.8	76.2 (0.5)	13.4 (0.4)	10.4 (0.4)	
3	2.9	65.1 (1.6)	16.8 (1.4)	18.1 (1.2)	

Values are presented as % (% standard error).

1 Chi-square test.

**Table 5.** Effects of mental health on risk of falls by fall status

Variables	Fall status	
	Single	Recurrent
Sleep duration (hr)		
≤5	1.01 (0.93, 1.10)	1.12 (1.02, 1.23)^*^
6-8	1.00 (reference)	1.00 (reference)
≥9	1.15 (0.99, 1.32)	1.24 (1.07, 1.44)^*^
Subjective stress level		
Rarely	1.00 (reference)	1.00 (reference)
A little	1.03 (0.95, 1.12)	1.21 (1.09, 1.34)^***^
Much	1.09 (0.98, 1.22)	1.82 (1.62, 2.04)^***^
Very much	1.06 (0.87, 1.29)	2.30 (1.90, 2.78)^***^
Depression		
No	1.00 (reference)	1.00 (reference)
Yes	1.27 (1.12, 1.44)^***^	1.56 (1.38, 1.76)^***^

Values are presented as odds ratio (95% confidence interval). Values adjusted for gender, age, education level, marital status, living arrangement, monthly income, residence type, locality, national basic livelihood recipient status, smoking, drinking, high intensity exercise, moderate intensity exercise, walking, body mass index, sleep duration, subjective stress level, depression, hypertension, diabetes, hyperlipidemia, arthritis, number of chronic illnesses, and subjective health status.

* p<0.05,

*** p <0.001.

## SUPPLEMENTARY MATERIALS

Korean version is available at http://www.e-epih.org/.
