# Supplementary material for: The effects of mental health on recurrent falls among elderly adults, based on Korean Community Health Survey data
Source: Epidemiol Health. 2020 Feb 2;42:e2020005. doi: 10.4178/epih.e2020005 (PMC7285425; doi:10.4178/epih.e2020005)
Supplement: Supplementary file 1 [file epih-42-e2020005-suppl.docx]

< Original Article>

**한국 노인에서 정신건강이 낙상 재발에 미치는 영향:**

**2015년 지역사회건강조사를 중심으로**

**The effects of mental health on recurrent falls among elderly adults,**

**based on Korean Community Health Survey data**

Kyung Hee Jo^1^, Jong Park^2^, So Yeon Ryu^2^

^1^Department of Public Health, Graduate School of Chosun University, Gwangju; South Korea

^2^Department of Preventive Medicine, Chosun University Medical School, Gwangju, South Korea

Corresponding author: Jong Park

Department of Preventive Medicine, Chosun University Medical School

309 Pilmun-daero, Dong-gu, Gwangju 61452, South Korea

E-mail: jpark@chosun.ac.kr

**ABSTRACT**

**Objectives:** This study aimed to identify the effect of mental health on frequency of falls (single and recurrent falls) among elderly adults.

**Methods:** Data were drawn from the 2015 Korean Community Health Survey. A chi-square test was conducted to compare differences in fall frequency according to health-related behaviors, chronic diseases, and mental health. Subsequently, multinomial logistic regression analysis was used to identify the effects of mental health on single and recurrent falls based on variables found to be significant in the chi-square test.

**Results:** Recurrent falls were found to be more risky than single falls. Depression was significantly related to single falls (odds ratio [OR], 1.27; 95% confidence interval [CI], 1.12 to 1.44). Depression (OR, 1.56; 95% CI, 1.38 to 1.76), sleep disorder (OR, 1.12; 95% CI, 1.02 to 1.23; OR, 1.24; 95% CI, 1.07 to 1.44), and subjective stress (OR, 2.30; 95% CI, 1.90 to 2.78) were significantly related to recurrent falls.

**Conclusions:** The study’s findings suggest that specialized fall prevention programs are needed to address different types of falls in elderly adults. To prevent recurrent falls, systematic treatment strategies and rehabilitation training must improve physical function and mental health.

Keywords: Accidental falls, Mental health, Depression, Aged, Korea Community Health Survey

**INTRODUCTION**

낙상은 넘어지거나 떨어져서 몸을 다치는 것으로 모든 연령에서 발생 가능하며, 특히 노인의 사고사 원인에서 교통사고 다음으로 높은 비중을 차지하고 있다[1]. 65세 이상 노인의 약 30%가 낙상을 경험하고, 그 중 50%가 낙상 재발을 경험하고 있어 낙상은 노인 건강을 위협하는 원인이 된다[2].

우리나라의 65세 이상 노인인구는 2018년 약738만명으로 전체 인구의 14.3%를 차지한다. 노인인구의 비율이 7% 이상일 경우 ‘고령화 사회’, 14% 이상일 경우 ‘고령사회’로 분류하고 있다. 우리나라는 이미 노인 인구 비중이 증가하여 ‘고령사회’에 진입하였고, 이와 같은 상황에서 노인의 건강을 위협하고 있는 낙상에 대한 문제가 더욱 커지고 있다[3].

노인의 신체는 노화로 인해 골밀도 저하 및 근육량 감소가 나타나고 작은 충격에도 골절상을 입기가 쉬우며, 낙상사고가 발생할 경우에는 골절, 타박상, 혈종, 부종, 합병증 등의 신체적 손상이 발생하기 쉽다[4]. 65세 이상 노인에서 낙상으로 인한 10만명 당 사망률은 2015년 18.6명, 2016년 20.3명, 2017년 21.1명으로 꾸준히 증가하고 있으며[5], 낙상으로 인한 입원환자는 2011년에 비해 2015년에서 약 12만 4천명으로 32% 증가하여 낙상노인의 사회·경제적 부담이 높아지고 있다[7]. 또한 낙상으로 인한 합병증이 발생할 경우는 치료기간이 더욱 장기화되고 의료비 지출이 증가하여 더 큰 경제적인 부담을 줄 수 있다[6].

낙상에 영향을 미치는 주요 위험 요인은 고령, 여성, 독거, 음주, 만성질환, 이전의 낙상경험, 삶의 질, 우울, 환경적 위험요인 등이 복합적으로 작용한다[8,9]. 최근엔 낙상에 영향을 미치는 주요한 요인으로 정신건강을 꼽고 있으며, 특히 우울감이 심한 노인에서 낙상 및 낙상재발 위험이 높게 나타났다[10]. 이처럼 노인 우울감은 인지기능을 저하시키고 그로 인해 인체 기능에 영향을 미쳐 낙상을 유발할 수 있다[11,12].

일반적으로 낙상은 갑작스러운 사고로 인해 발생하므로 개입을 통한 예방이 어려운 경우가 많다[13]. 반면 재발성 낙상은 나이가 많고 연약한 노인 중 여러 합병증이나 정신적․생리적 문제를 가진 노인이 많으며[13], 낙상을 경험한 후에는 다시 낙상이 일어날지도 모른다는 불안감과 두려움으로 인해 스스로 일상생활을 제한하여 운동기능이 저하되어 낙상이 재발된다[14]. 이처럼 낙상은 1회낙상과 반복낙상이 서로 다른 기전을 가지고 있으므로, 낙상의 발생 빈도에 따라 1회낙상자와 반복낙상자를 구분하여 비교하는 것은 매우 중요하다[14].

노인 낙상에 영향을 미치는 요인을 분석한 연구들은 다수 보고되고 있다. 국내의 연구들은 주로 일부 소규모 집단을 대상으로 낙상 평가 모형을 사용하여 위험요인을 확인하였고[15,16,17], 정신건강연구는 우울에서 낙상에 미치는 영향을 확인한 연구만 있어[18,19], 정신건강이 낙상에 미치는 영향을 설명하기에 충분한 연구가 이루어지지 않은 실정이다.

이에 본 연구는 지역사회기반의 데이터를 사용하여 노인에서 연간 낙상 발생 빈도에 따라 1회낙상군과 반복낙상군(2회 이상)으로 구분하여, 그 특성을 파악하고, 그를 통해 정신건강이 1회낙상과 반복낙상에 미치는 영향을 비교하고자 한다.

**MATERIALS AND METHODS**

1.연구자료 및 대상

본 연구는 질병관리본부가 주관하는 2015년 지역사회건강조사 원시자료를 이용하였다. 각 지역에 거주하는 만 19세 이상 성인을 모집단으로 하여 확률비례 계통추출법을 적용하여 표본지점을 선정하고, 선정된 표본지점에서 계통추출법에 의해 가구를 선정한 후, 전국의 254개 보건소에서 약 900명의 표본자료를 수집한다. 조사시점 표본가구에 거주하는 만 19세 이상 성인을 대상으로 2015년 8월 31일부터 11월 8일까지 훈련된 조사원이 표본으로 선정된 가구에 직접 방문하여 설문프로그램이 탑재된 노트북을 사용하여 19개 영역의 198개 문항을 1:1면접조사(전자설문조사)를 통해 자료를 수집하였다[20].

본 연구는 2015년 지역사회건강조사 조사대상자 전체 228,558명에서 65세 이상인 63,929명 중 연간 낙상경험과 낙상 건수 문항에서 응답이 불충분한 30명을 제외한 63,899명을 대상자로 선정하였다.

2.연구 내용 및 변수

65세 이상 노인에서 낙상에 영향을 미치는 요인을 파악하기 위해 인구사회학적 요인, 건강행태, 신체건강관련 요인, 정신건강관련 요인을 사용하였다.

1) 낙상관련 요인

낙상은 연간 낙상 경험 여부에서 예라고 응답한 대상자 중에서 연간 낙상 건수에 따라 낙상경험 ‘없음’, ‘한번’, ‘두 번 이상’으로 구분하였다. 낙상에 대한 두려움은 “평소 넘어지는 것에 대한 두려움을 느낍니까?”에서 ‘전혀 두려워하지 않는다’, ‘약간 두려워한다’, ‘많이 두려워한다’로 구분하였다. 연간 낙상치료경험은 “최근 1년 동안 넘어진 적이 있습니까?”에서 예, 아니오로 구분하였다. 침상와병경험 여부는 “최근 1달 동안 질병이나 손상으로 거의 하루 종일 누워서 보내야 했던 적이 있습니까?”에서 예, 아니오로 구분하였다.

2) 인구사회학적 요인

인구사회학적 요인은 성별, 연령, 학력, 배우자 유무, 거주유형, 월수입, 주택유형, 지역구분, 기초생활수급자 여부를 사용하였다. 성별은 남자, 여자로 구분하였다. 학력은 최종적으로 다닌 학교와 졸업여부에 따라 무학, 초등학교, 중학교, 고등학교, 대학교 이상으로 분류하였다. 배우자유무는 배우자 있음에 해당되는 경우 ‘있다’ 이혼, 사별, 별거, 미혼인 경우는 ‘없다’로 분류하였다. 거주유형은 독거, 부부, 그 외로 구분하였고, 월수입은 100만원 미만, 100-200만원 미만, 200-300만원미만, 300만원이상으로 분류하였다. 주택유형은 주택과 아파트로 구분하였고, 지역구분은 행정구역상 동단위에 거주하는 경우 ‘도시’ 읍‧면 단위에 거주하는 경우는 ‘농촌’으로 분류하였다. 기초생활수급자여부는 “현재 기초생활 수급자 여부”에 따라 예, 아니오로 분류하였다.

3) 건강행태 요인

흡연은 현재흡연여부에서 매일피움 또는 가끔피움으로 응답한 경우 “예”, 과거에는 피웠으나 현재 피우지 않음 또는 비해당은 “아니오”로 구분하였다. 음주는 음주빈도에 따라 비음주(한달에 1번미만), 적정음주(한달에1-4번정도), 고위험음주(일주일에 2번이상)로 구분하였고, 특히 고위험음주는 일주일에 2번이상 술을 마시며 한번에 남자 소주7잔, 여자 소주5잔 이상을 마시는 경우로 정의하였다. 격렬한 신체활동은 20분 이상 3일 이상 한 경우, 중강도 신체활동은 30분 이상 주5일이상 한 경우, 걷기는 30분이상 주5일 이상 한 경우를 ‘예’로 구분하였다. 주관적 건강수준은 “평소에 본인의 건강은 어떻다고 생각합니까?”에서 좋음, 보통, 나쁨으로 구분하였다. BMI는 키와 몸무게를 이용해 체질량지수(Body Mass Index)를 산출하였고, 저체중(18.5𝑘𝑔/𝑚^2^미만), 적정체중(18.5𝑘𝑔/𝑚^2^이상 25.0𝑘𝑔/𝑚^2^미만), 비만(25.0𝑘𝑔/𝑚^2^이상 30.0𝑘𝑔/𝑚^2^미만), 고도비만(30.0𝑘𝑔/𝑚^2^이상)으로 구분하였다.

4) 만성질환관련 요인

고혈압/ 당뇨병/ 이상지질혈증/ 관절염은 현재치료여부에 따라 예, 아니오로 구분하였다. 만성 질환 수는 고혈압, 당뇨병, 관절염 중에서 현재 치료 중인 질병의 수에 따라 0개, 1개, 2개, 3개로 구분하였다.

5) 정신건강관련 요인

주관적 스트레스는 “평소 일상생활 중에서 스트레스를 어느 정도 느끼고 있습니까?”에서 대단히 많이 느낀다, 많이 느끼는 편이다, 조금 느끼는 편이다, 거의 느끼지 않는다 로 구분하였고, 우울감 경험여부는 “최근 1년 동안 연속적으로 2주 이상 일상생활에 지장이 있을 정도로 슬프거나 절망감 등을 느낀 적이 있습니까?”에서 예, 아니오로 구분하였다. 수면시간은 6시간 미만, 9시간 이상, 6-8시간으로 구분하였고, 6시간 미만 및 9시간 이상을 수면장애, 6-8시간은 정상수면으로 정의하였다[21].

3.분석방법

통계 분석도구는 IBM SPSS Statistics 25.0 프로그램을 사용하였다. 분석에 사용된 독립변수를 선정하기위해 기존 낙상관련 연구의 문헌고찰을 통해 낙상에 영향을 미칠 수 있는 요인들을 독립변수로 선정하였다. 통계분석을 위해 복합표본설계를 고려하고, 모집단을 추정하기 위해 개인 가중치를 적용하여 분석하였다. 1회낙상군과 반복낙상군의 변수간의 차이를 확인하기 위해 복합표본 교차분석 카이제곱 검정(chi-square test)을 실시하였고, 각 군의 관련 요인의 차이를 밝히기 위해 65세 이상을 모집단으로 정의하고 낙상빈도를 종속변수로 설정하여 복합표본 로지스틱 회귀분석(multinominal logistic regression)모델을 이용하여 위험비(odds ratio, OR)를 산출하였다. 통계 검정을 위한 유의수준은 0.05 미만으로 설정하였다.

**RESULTS**

1.대상자의 낙상 관련 특성

65세 이상 노인의 연간 낙상경험여부에서 경험자는 19.6%, 비 경험자는 80.4%이다. 연간 낙상 건수는 1회 경험자는 11.6%, 2회 이상 경험자는 8.0%이었다. 연간 낙상치료 경험 여부는 치료를 받은 군은 48.1%, 치료를 받지 않은 군은 51.9%이었다(Table 1). 낙상에 대한 두려움은 ‘전혀 두려워하지 않는다’는 23.5% ‘약간 두려워한다’는 37.7% ‘많이 두려워한다’는 38.8%이었고, 낙상에 대한 두려움이 가장 많은 군에서 낙상경험과 반복낙상 비율이 높았다. 침상와병 경험 여부에서는 경험자가 91.9%, 비경험자가 8.1%이었고, 침상와병 경험이 있는 군에서 낙상과 반복낙상 비율이 높게 나타났다(Table 2).

2.낙상횟수별 대상자의 일반적 특성과 건강행태 특성

연구대상자는 성별에서 남자가 여자보다 낙상 경험과 반복낙상 비율이 높았고, 연령은 비 낙상군이 73.1세, 1회낙상군 73.9세, 반복낙상 74.8세로 연령이 증가할수록 낙상발생빈도가 높았다. 배우자 유무는 배우자가 없는 경우 낙상발생이 높았으며, 거주유형은 독거노인인 경우 부부가 거주하는 경우보다 낙상을 경험한 비율이 높았다. 거주지역은 도시에서 1회낙상 발생이 높았고, 농촌에서 반복낙상 발생이 높았으며, 기초생활수급자인 경우 낙상경험과 반복낙상 비율이 높았다. 흡연은 비흡연자에서 낙상경험과 반복낙상 비율이 높았고, 음주는 비음주자에서 낙상경험과 반복낙상 비율이 높았다. 고강도운동/중강도운동/걷기의 신체활동을 하지 않는 사람의 경우 낙상경험과 반복낙상 비율이 높았으며, 주관적 건강수준은 나쁨일 때 낙상경험과 반복낙상 비율이 높았다. 체질량지수는 고도비만에서 낙상경험 비율이 높았고, 저체중과 고도비만에서 반복낙상 비율이 높았다. 언급된 모든 변수들은 통계적으로 유의한 차이가 있었다(Table 3).

3.낙상횟수별 정신건강과 만성질환 특성

정신건강에서는 수면 시간이 5시간 미만 또는 9시간 이상일 경우 6-8시간보다 1회낙상과 반복낙상의 경험 비율이 높았으며, 주간적 스트레스는 스트레스 수준이 높아질수록 1회낙상과 반복낙상 비율이 높았다. 우울감 경험은 우울감을 경험한 경우 1회낙상과 반복낙상 비율이 높았다. 만성질환에서는 고혈압/당뇨병/이상지질혈증/관절염을 현재 치료중인 경우 1회낙상과 반복낙상 비율이 높았고, 대상자가 가지고 있는 만성질환 수가 많을수록 1회낙상과 반복낙상 비율이 높았다. 주관적 건강수준은 평소 본인의 건강에 대해 나쁘다고 생각할수록 1회낙상과 반복낙상 비율이 높았다. 정신건강과 만성질환 관련 모든 변수는 통계적으로 유의한 차이가 있었다(Table 4).

4.정신건강이 낙상 빈도에 미치는 영향

다항 로지스틱 회귀분석을 실시한 결과 1회낙상군의 경우 우울감을 경험한 경우 낙상위험이 1.27(95% CI: 1.12-1.44)로 높았다. 반복낙상군의 경우 우울감을 경험했을때 반복낙상위험이 1.56(95% CI: 1.38-1.76)로 더 높았고, 수면시간이 6시간미만일때 반복낙상위험이 1.12(95% CI:1.02-1.23), 9시간 이상일때 1.24(95% CI: 1.07-1.44)로 높았다. 주관적 스트레스에서는 스트레스를 조금 느끼는 경우 1.21(95% CI: 1.09-1.34), 대단히 많이 느끼는 경우 2.30(95% CI: 1.90-2.78)으로 스트레스를 많이 느끼는 경우 반복낙상 위험이 더 크게 나타났다(Table 5).

**DISCUSSION**

노인의 건강상태는 낙상에 영향을 미칠 수 있으며, 신체적 건강뿐만 아니라 정신적 건강과도 밀접한 관련성이 있다. 건강상태가 취약한 노인일수록 단순 낙상이 반복낙상으로 연결되고 노인 건강에 치명적인 영향을 미치게 되며 사망까지 이르게 할 수 있다[13].

우리나라 65세 이상 노인 중 지난 1년간 낙상을 경험한 비율은 약 19.6%이다. 그 중 연간 ‘1회낙상군’은 59.2%, 2회 이상의 낙상을 경험한 ‘반복낙상군’은 40.8% 이다. Jeon&Yang[22]의 연구는 농촌지역에 거주하는 재가노인 329명에서 1회낙상자가 73.3%, 낙상재발자가 21.7%을 차지했고, Yoo[15]의 연구에서는 일부 지역 보건소와 복지회관에 등록된 재가노인 104명에서 1회낙상자가 55.7%, 반복낙상자가 44.2% 수준이었다. 기존 연구는 일부 집단을 대상으로 연구하여 낙상자의 특성에 차이가 있으며, 이는 연구결과에도 영향을 줄 수 있으므로 한국 노인의 낙상을 규명하기에는 어려움이 있었다. 이에 본 연구는 2015년 지역사회건강조사 자료를 이용하여 낙상경험 빈도에 따라 ‘1회낙상군’과 ‘반복낙상군’으로 구분하여 노인의 정신건강이 낙상에 미치는 영향을 확인하였다.

본 연구의 결과는 ‘1회낙상군’은 우울감 경험을 한 경우에서 낙상 위험이 증가했고, ‘반복낙상군’은 우울감을 느끼고, 수면장애가 있으며(수면시간 6시간 미만, 9시간 이상), 주관적 스트레스가 커질수록 낙상 위험이 증가하였다. 따라서 노인의 정신건강에 반복낙상이 미치는 영향이 더 큰 것을 확인하였다. 반복낙상자는 낙상을 경험한 후 겪게되는 낙상두려움으로 인해 일상생활에서 신체활동이 감소하게 되고 정서적인 부분에도 영향을 주어 반복낙상을 유발할 수 있는 요인이 될 수 있다[15].

우울감경험은 1회낙상군에서 낙상 위험이 1.27배 유의하게 증가하였고, 국외연구의 경우 노인 우울과 낙상의 연관성을 분석한 결과에서 우울증상이 높아질수록 낙상 위험이 높아졌고[23], 국내에서도 우울증이 있는 그룹에서 낙상 발생 위험이 증가하는 결과를 확인하였다[19]. 우울증은 노인의 정신적인 기능을 떨어뜨리고 그로 인해 인지기능 저하를 초래하여 신체 기능에도 영향을 미치게 하므로, 이러한 기전에 영향을 받았을 것으로 사료된다[11,12]. 반복낙상군에서는 우울감의 낙상 재발위험이 1.56배 증가하여 1회낙상군에 비해 위험이 더 크게 나타났다. 반복낙상은 우울과 밀접한 상관관계가 있고[21], 우울증상을 가진 환자의 경우 낙상 재발률이 45% 더 높았다[24]. 또한, 1회낙상을 경험한 후, 육체적인 손상으로 인해 신체기능, 사회활동이 감소하고, 그로 인해 우울감이 증가하여 재발낙상을 유발시키는 주요한 요인이 된다[18].

그 외에도 정신건강은 수면의 시간이나 질 과도 밀접한 관련성이 있으며, 통상적으로 수면시간이 6시간 미만 혹은 9시간 이상인 경우 수면 장애로 정의된다[21]. 본 연구에서 반복낙상군에서

수면장애를 가진 경우 낙상 재발위험이 커지는 경향을 보였으며, 특히 9시간 이상의 과다수면의서 반복낙상 위험이 더 커지는 결과를 확인하였다. 일부 연구에서는 과다수면(9시간 이상 수면)인 경우 수면부족(5시간 이하 수면)보다 수면의 질을 더 떨어뜨릴 수 있다고 보고된다[25]. 또한, 수면의 질 저하는 신체적 건강기능을 저하시키고 정신적 인지기능에 영향을 미치게 되어 결국 낙상에 영향을 주게 될 수 있고[26], 이미 낙상을 경험한 노인일 경우 재발성 낙상위험도 증가하게 된다[27]. 그러나 수면상태를 정확하게 평가하기 위해서는 수면의 양, 수면 시 각성횟수, 수면까지 걸리는 시간 등 수면에 대한 다양한 부분을 고려해야 하므로[28], 단순히 수면시간으로 수면의 상태를 평가하기에는 어려움이 있다. 또한 수면상태를 평가하여 재발성 낙상에 미치는 영향을 보고한 연구결과가 매우 드문 실정이므로 추후 후속 연구가 필요할 것으로 생각된다.

주간적 스트레스 결과에서도 반복낙상군이 스트레스가 높아질수록 낙상 재발 위험이 커지는 결과를 확인하였다. 1회 낙상은 갑작스러운 사고로 인해 발생하지만, 반복낙상은 재발되는 낙상경험으로 인해 낙상에 대한 두려움을 증가시켜 심리적인 스트레스를 가중시킴으로써 발생한다[13]. 결국 이와 같은 부정적인 영향은 신체적 활동 능력과 기능 회복에도 영향을 미친다[29]. 그러므로 반복낙상을 경험한 노인의 경우 단순히 신체적 기능 회복을 위한 훈련뿐만 아니라 자기 효능감을 높이고 스트레스를 완화시킬 수 있는 정신건강 증진 프로그램을 병행함이 필요할 것으로 생각된다.

본 연구는 우리나라 노인을 대상으로 한 단면연구로 정신건강이 낙상에 미치는 영향을 확인하였고, 반복낙상에서 영향력이 더 커지는 것을 확인하였다. 이와 같은 영향은 여러 복합적인 요인들이 정서적인 부분에 영향을 미쳤을 것으로 생각된다. 그로 인해 스트레스와 우울감을 경험하여 수면시간이 길어지는 결과를 유도했을 것으로 생각되나, 정신건강변수간의 선후관계를 설명하기에는 어려움이 있었다. 또한, 반복낙상과 정신건강의 관계에 있어 방향성을 설명하는데 어려움이 있다는 제한점이 있다. 그럼에도 본 연구는 지역사회에 기반한 대표성 있는 데이터베이스를 사용하였고, 정신건강이 1회낙상과 반복낙상에 미치는 영향 밝히고 그 차이를 비교했다는 점에서 의의가 있다.

**CONCLUSION**

한국 노인은 스트레스증가, 우울감 경험, 수면장애로 인해 정신적 건강이 취약할수록 반복낙상 위험이 높아질 수 있다. 1회낙상의 경우 갑작스러운 사고로 인해 발생하지만, 반복낙상은 고위험군에서 주로 발생하므로 예방을 통한 낙상 빈도 감소가 가능하다. 그러므로 낙상 경험 빈도에 따라 위험요인을 관리하고 차별화된 낙상예방 프로그램이 필요하며, 특히 반복낙상자의 경우 정신건강과 신체적 기능을 함께 증진시킬 수 있는 체계적인 치료 전략과 재활 훈련이 필요할 것이다.

**REFERENCES**

1. World Health Organization. The injury chartbook. 2002, p. 6-18.

2. Ruchinskas R. Clinical prediction of falls in the elderly. Arch Phys Med Rehab 2003;82:273-278.

3. Statistics Korea (KOSTAT). 2018 Elderly statistics [in Korean]. 2018, p. 3-4.

4. Ryeom TH, Kim SY, So YK, Park SY, Lee JH, Cho HS, et al. The risk factors of falls in the elderly. Korean J Fam Med 2001;22:221-229 (Korean).

5. Statistics Korea(KOSTAT). Annual report on the cause of death statistics. 2017,p19-22 (Korean).

6. Tinetti ME, Speechley M, Ginter SF. Risk factors for falls among elderly persons living in the community. N Engl J Med 1988;319:1701-1707.

7. Korea Centers for Disease Control and Prevention(KCDC). National Injury Fact Book 2015-2016. 2018,p.79-80 (Korean, author’s translation).

8. Rubenstein LZ, Josephson KR. Falls and Their Prevention in Elderly People: What Does the Evidence Show? Med Clin North Am 2006;90:807-824.

9. Letts L, Moreland J, Richardson J, Coman L, Edwards M, Ginis KM, et al. The physical environment as a fall risk factor in older adults: systematic review and meta-analysis of cross-sectional and cohort studies. Aust Occup Ther J 2010;57: 51-64.

10. Yeom JH, Na HJ. Risk factors of falls among Korean elderly. Gerontological Society 2012;32:577-592 (Korean).

11. Turcu A, Toubin S, Mourey F, D’Athis P, Manckoundia P, Pfitzenmeyer P. Falls and depression in older people. Gerontology 2004;50:303-308.

12. Rock PL, Roiser JP, Riedel WJ, Blackwell AD. Cognitive impairment in depression: a systematic review and meta-analysis. Psychol Med 2014;44:2029-2040.

13. Fletcher PC, Hirdes JP. Risk factors for falling among community-based seniors using home care services. J Gerontol:Medical Sciences 2002;57:504-510

14. Hong SH, Cho EH, Choi MY. Risk factors for falls among community-dwelling older adults: Using KLoSA data. J Korean Gerontol Nurs 2010;12:211-224 (Korean).

15. Yoo IY. Analysis of multi-variate recurrent fall risk factors in elderly people using residential assessment instrument-home care: Comparisons between single and recurrent fallers. J Korean Acad Nurs 2011;41:119-128 (Korean).

16. Kang KS, Yang J. A study on fear of falling, activity of daily living and quality of life for the elderly. Academia-industrial 2017;.18:193-199 (Korean).

17. Lee HJ, Lee TY, Tae KS. A study on the prediction of fall factors for the elderly living in the city. rehabilitation welfare engineering&assistive technology 2018;12:46-52 (Korean).

18. Nam IS, Yoon HS. An analysis of the interrelationship between depression and falls in Korean older people. Gerontological Society 2014;34:523-537 (Korean).

19. Kim SG, Kim SJ, Kim KH, Jeong SM, Ko AR, Han SV, et al. The Association between Depression and Falls in Korean Adults Aged 45 Years or Older. Korean J Fam Pract 2018;8:696-702 (Korean).

20. Korea Centers for Disease Control and Prevention(KCDC). Community health survey 2015. 2015,p4-7 (Korean, author’s translation).

21. Kim MS, KimJI. Relationship among the health state, daily living activeties(ADL, IADL), sleep state, and depression among old people at elderly care facilities. Academia-industrial 2015;16:2609-2619.

22. Jeon MY, Yang SH. Risk factors for recurrent falls among community-dwelling elderly in rural areas. Academy Industrial Cooperation Society 2013;14:6353-6363 (Korean).

23. Kvelde T, McVeigh C, Toson B, Greenaway M, Lord SR, Delbaere K, et al. Depressive symptomatology as a risk factor for falls in older people: systematic review and meta-analysis. J Am Geriatr Soc 2013;61:694-706.

24. Marcum ZA, Perera S, Thorpe JM, Switzer GE, Castle NG, Strotmeyer ES, et al. Antidepressant use and recurrent falls in community-dwelling older adults: findings from the Health ABC Study. Ann Pharmacother 2016;50:525-33.

25. Kwon EJ, Kim BS, Won CW, Choi HR, Kim SY, Kim KJ, et al. The effect of sleep duration and regularity on cardio-cerebrovascular disease: community based prospective study. Korean J Fam Pract 2018;8:729-734 (Korean).

26. Ancoli-Israel S. Sleep and its disorders in aging populations. Sleep Medicine 2009;10:7-11.

27. Shin KR, Gong SJ, Kang Y, Oak JW, Lim EJ. The relationship of late-life function anddisability(LLFDI) with quality of sleep in older men with prostatic hypertrophic symptoms. Adult Nursing 2009;21,43-52 (Korean).

28. Pilcher JJ, Ginter DR, Sadowsky B. Sleep quality versus sleep quantity: Relationship between sleep and measures of health, well-being and sleepiness in college students. J Psychosom Res, 1997;42:583-596.

29. Park CS, Kim WG, An　SH. The study on predicting of fall incidence using the falls-related efficacy scale in people with chronic Stroke. Special Education & Rehabilitation Science 2013;52:241-258 (Korean).

Table 1. Fall-related characteristics of adults aged 65 and older

| **Variable** | ***N*** | **Estimated**  **value (%)** | **Standard**  **error (%)** |
| --- | --- | --- | --- |
| **Fall experience** | 63,899 |  |  |
| Yes | 12,659 | 19.6 | 0.21 |
| No | 51,240 | 80.4 | 0.21 |
| **Number of falls in the past year** | 63,899 |  |  |
| 0 | 51,240 | 80.4 | 0.21 |
| 1 | 7,190 | 11.6 | 0.18 |
| ≥ 2 | 5,469 | 8.0 | 0.15 |
| **Treatment for a fall within the past year** | 12,659 |  |  |
| Yes | 5,972 | 48.1 | 0.62 |
| No | 6,687 | 51.9 | 0.62 |

Table 2. Fall-related characteristics according to fall status in adults aged 65 and older

| **Variable** | **Total** | **No falls** | **Single fall** | **Recurrent falls** | ***p*-value**^a^ |
| --- | --- | --- | --- | --- | --- |
|  | **%** | **% (% SE)** | **% (% SE)** | **% (% SE)** |  |
| **Fear of falling** |  |  |  |  |  |
| Not at all afraid | 38.8 | 90.4 (0.3) | 6.8 (0.2) | 2.8 (0.1) | < 0.001 |
| A little afraid | 37.7 | 81.1 (0.4) | 11.9 (0.3) | 7.0 (0.2) |  |
| Very afraid | 23.5 | 62.8 (0.5) | 19.0 (0.4) | 18.1 (0.4) |  |
| **Bedridden in past month** |  |  |  |  |  |
| Yes | 8.1 | 61.8 (0.9) | 17.2 (0.7) | 21.0 (0.8) | < 0.001 |
| No | 91.9 | 82.1 (0.2) | 11.1 (0.2) | 6.8 (0.1) |  |

^a^ Chi-square test.

Table 3. Differences in general characteristic and health behaviors by fall status in adults aged 65 and older

| **Variable** | **Total** | **No falls** | **Single fall** | **Recurrent falls** | | ***p*-value**^a^ | | |  |
| --- | --- | --- | --- | --- | --- | --- | --- | --- | --- |
|  | **%** | **% (% SE)** | **% (% SE)** | **% (% SE)** | |  |  |  |  |
| **General information (*N*)** | **63,899** | **51,240** | **7,190** | **5,469** | |  | | |  |
| **Gender** |  |  |  |  | | < 0.001 | | |  |
| Male | 43.2 | 76.3 (0.3) | 14.0 (0.2) | 9.7 (0.2) | |  |  |  |  |
| Female | 56.8 | 85.9 (0.2) | 8.4 (0.2) | 5.7 (0.2) | |  |  |  |  |
| **Age (years)** |  |  |  |  | |  | | |  |
| Mean ± SE | 73.91 ± 0.06 | 73.08 ± 0.04 | 73.85 ± 0.10 | 74.80 ± 0.12 | | < 0.001 | | |  |
| **Education level** |  |  |  |  | | < 0.001 | | |  |
| No formal education | 26.0 | 87.2 (0.7) | 9.1 (0.6) | 3.8 (0.4) | |  |  |  |  |
| Elementary school | 31.1 | 84.8 (0.5) | 9.3 (0.4) | 5.9 (0.3) | |  |  |  |  |
| Middle school | 15.4 | 83.2 (0.6) | 11.2 (0.5) | 5.6 (0.3) | |  |  |  |  |
| High school | 19.4 | 84.8 (0.5) | 9.3 (0.4) | 5.9 (0.3) | |  |  |  |  |
| College or higher | 8.1 | 87.2 (0.7) | 9.1 (0.6) | 3.8 (0.4) | |  |  |  |  |
| **Marital status** |  |  |  |  | | < 0.001 | | |  |
| No spouse | 35.8 | 75.4 (0.4) | 14.0 (0.3) | 10.6 (0.3) | |  |  |  |  |
| Spouse | 64.2 | 83.2 (0.3) | 10.3 (0.2) | 6.5 (0.2) | |  |  |  |  |
| **Living arrangement** |  |  |  |  | | < 0.001 | | |  |
| Alone | 19.1 | 75.3 (0.5) | 13.7 (0.4) | 11.0 (0.3) | |  |  |  |  |
| With spouse | 43.8 | 83.4 (0.3) | 10.1 (0.2) | 6.5 (0.2) | |  |  |  |  |
| With family or others | 37.1 | 79.5 (0.4) | 12.3 (0.3) | 8.2 (0.3) | |  |  |  |  |
| **Monthly income (KRW)** |  |  |  |  | | < 0.001 | | |  |
| < 1 million | 43.2 | 77.3 (0.3) | 12.7 (0.3) | 10.0 (0.2) | |  |  |  |  |
| 1-2 million | 22.4 | 82.5 (0.4) | 10.9 (0.4) | 6.5 (0.3) | |  |  |  |  |
| 2-3 million | 14.0 | 83.3 (0.6) | 10.7 (0.5) | 6.0 (0.4) | |  |  |  |  |
| ≥ 3 million | 20.4 | 82.7 (0.5) | 10.7 (0.4) | 6.5 (0.3) | |  |  |  |  |
| **Residence type** |  |  |  |  | | < 0.001 | | |  |
| House | 64.7 | 81.3 (0.4) | 11.7 (0.3) | 7.0 (0.3) | |  |  |  |  |
| Apartment | 35.3 | 79.9 (0.2) | 11.6 (0.2) | 8.5 (0.2) | |  |  |  |  |
| **Locality** |  |  |  |  | | < 0.001 | | |  |
| Urban | 69.9 | 80.6 (0.3) | 11.8 (0.2) | 7.6 (0.2) | |  |  |  |  |
| Rural | 30.1 | 80.1 (0.3) | 11.0 (0.2) | 8.9 (0.2) | |  |  |  |  |
| **National basic livelihood recipient status** | | | | |  |  |  |  | |
| Yes | 6.3 | 70.9 (1.0) | 15.1 (0.8) | 14.0 (0.7) | | < 0.001 | | |  |
| No | 98.7 | 81.1 (0.2) | 11.4 (0.2) | 7.6 (0.1) | |  | | |  |
| **Smoking** |  |  |  |  | | < 0.001 | | |  |
| Yes | 9.3 | 84.6 (0.6) | 9.4 (0.5) | 5.9 (0.4) | |  |  |  |  |
| No | 90.7 | 80.0 (0.2) | 11.8 (0.2) | 8.2 (0.2) | |  |  |  |  |
| **Drinking** |  |  |  |  | | < 0.001 | | |  |
| None | 65.9 | 78.8 (0.3) | 12.4 (0.2) | 8.9 (0.2) | |  |  |  |  |
| Low to moderate | 23.2 | 83.3 (0.4) | 10.2 (0.4) | 6.5 (0.5) | |  |  |  |  |
| High-risk | 10.9 | 85.0 (0.6) | 9.6 (0.5) | 5.4 (0.4) | |  |  |  |  |
| **High intensity exercise** |  |  |  |  | |  | | |  |
| Yes | 9.8 | 83.6 (0.7) | 9.9 (0.6) | 6.5 (0.4) | | < 0.001 | | |  |
| No | 90.2 | 80.1 (0.2) | 11.8 (0.2) | 8.1 (0.2) | |  |  |  |  |
| **Moderate intensity exercise** |  |  |  |  | | < 0.001 | | |  |
| Yes | 19.0 | 83.5 (0.4) | 10.1 (0.4) | 6.4 (0.3) | |  |  |  |  |
| No | 81.0 | 79.7 (0.2) | 12.0 (0.2) | 8.3 (0.2) | |  |  |  |  |
| **Walking** |  |  |  |  | | < 0.001 | | |  |
| Yes | 51.6 | 83.0 (0.3) | 10.7 (0.3) | 6.3 (0.2) | |  |  |  |  |
| No | 48.4 | 77.7 (0.3) | 12.6 (0.2) | 9.7 (0.2) | |  |  |  |  |
| **Subjective health status** |  |  |  |  | | < 0.001 | | |  |
| Good | 21.6 | 88.7 (0.4) | 8.1 (0.3) | 3.3 (0.2) | |  |  |  |  |
| Average | 36.5 | 83.5 (0.3) | 11.2 (0.3) | 5.3 (0.2) | |  |  |  |  |
| Poor | 41.9 | 73.5 (0.4) | 13.8 (0.3) | 12.7 (0.3) | |  |  |  |  |
| **BMI (kg/m^2^)** |  |  |  |  | | < 0.001 | | |  |
| Underweight (≤18.5) | 12.3 | 77.0 (0.6) | 12.5 (0.4) | 10.6 (0.4) | |  |  |  |  |
| Healthy weight (18.5-25.0) | 64.6 | 81.4 (0.3) | 11.4 (0.2) | 7.2 (0.2) | |  |  |  |  |
| Obese (25.0-30.0) | 21.3 | 80.3 (0.5) | 11.3 (0.4) | 8.5 (0.5) | |  |  |  |  |
| Severely obese (≥30.0) | 1.8 | 74.2 (2.1) | 15.2 (1.8) | 10.6 (1.5) | |  |  |  |  |

*Note:* BMI, body mass index; KRW, Korean won.

^a^Chi-square test.

Table 4. Differences in mental health and chronic illness characteristics by fall status in adults aged 65 and older

| **Variable** | **Total** | **No falls** | **Single fall** | **Recurrent falls** | ***p*-value**^a^ |
| --- | --- | --- | --- | --- | --- |
|  | **%** | **% (% SE)** | **% (% SE)** | **% (% SE)** |  |
| **Sleep duration (hours)** |  |  |  |  |  |
| ≤ 5 | 25.5 | 77.5 (0.4) | 12.4 (0.4) | 10.1 (0.3) | < 0.001 |
| 6-8 | 68.7 | 81.8 (0.3) | 11.2 (0.2) | 7.0 (0.2) |  |
| ≥ 9 | 5.8 | 76.7 (0.9) | 13.2 (0.7) | 10.1 (0.6) |  |
| **Subjective stress level** |  |  |  |  |  |
| Rarely | 35.1 | 83.3 (0.3) | 10.9 (0.3) | 5.7 (0.2) | < 0.001 |
| A little | 44.7 | 81.6 (0.3) | 11.5 (0.3) | 6.9 (0.2) |  |
| Much | 17.3 | 73.5 (0.6) | 13.1 (0.5) | 13.4 (0.5) |  |
| Very much | 2.9 | 67.7 (1.4) | 13.1 (1.0) | 19.3 (1.2) |  |
| **Depression** |  |  |  |  |  |
| Yes | 8.3 | 67.7 (0.9) | 15.1 (0.7) | 17.2 (0.8) | < 0.001 |
| No | 91.7 | 81.3 (0.2) | 11.3 (0.2) | 7.1 (0.1) |  |
| **Hypertension** |  |  |  |  |  |
| Yes | 51.0 | 79.3 (0.3) | 12.0 (0.2) | 8.7 (0.2) | < 0.001 |
| No | 49.0 | 81.5 (0.3) | 11.2 (0.3) | 7.2 (0.2) |  |
| **Diabetes** |  |  |  |  |  |
| Yes | 20.3 | 77.2 (0.5) | 13.3 (0.4) | 9.6 (0.4) | < 0.001 |
| No | 79.7 | 81.3 (0.2) | 11.2 (0.2) | 7.6 (0.2) |  |
| **Hyperlipidemia** |  |  |  |  |  |
| Yes | 18.4 | 77.7 (0.6) | 13.0 (0.4) | 9.2 (0.4) | < 0.001 |
| No | 81.6 | 81.1 (0.2) | 11.3 (0.2) | 7.7 (0.2) |  |
| **Arthritis** |  |  |  |  |  |
| Yes | 18.7 | 71.2 (0.5) | 14.7 (0.4) | 14.1 (0.4) | < 0.001 |
| No | 81.3 | 82.6 (0.2) | 10.9 (0.2) | 6.6 (0.1) |  |
| **Number of chronic illnesses** | | | | |  |
| 0 | 35.7 | 83.8 (0.3) | 10.3 (0.3) | 5.9 (0.2) | < 0.001 |
| 1 | 41.5 | 80.6 (0.3) | 11.5 (0.3) | 7.9 (0.2) |  |
| 2 | 19.8 | 76.2 (0.5) | 13.4 (0.4) | 10.4 (0.4) |  |
| 3 | 2.9 | 65.1 (1.6) | 16.8 (1.4) | 18.1 (1.2) |  |

^a^Chi-square test.

Table 5. Effects of mental health on risk of falls by fall status

| **Variable** | | **Single fall/No falls** | | **Recurrent falls/No falls** | |
| --- | --- | --- | --- | --- | --- |
|  |  | **OR** | **95% CI** | **OR** | **95% CI** |
| **Sleep duration (hours)** |  |  |  |  |  |
|  | ≤ 5 | 1.01 | 0.93-1.10 | 1.12 | 1.02-1.23* |
|  | 6-8 | 1.00 | Reference | 1.00 | Reference |
|  | ≥ 9 | 1.15 | 0.99-1.32 | 1.24 | 1.07-1.44* |
| **Subjective stress level** |  |  |  |  |  |
|  | Rarely | 1.00 | Reference | 1.00 | Reference |
|  | A little | 1.03 | 0.95-1.12 | 1.21 | 1.09-1.34** |
|  | Much | 1.09 | 0.98-1.22 | 1.82 | 1.62-2.04** |
|  | Very Much | 1.06 | 0.87-1.29 | 2.30 | 1.90-2.78** |
| **Depression** |  |  |  |  |  |
|  | No | 1.00 | Reference | 1.00 | Reference |
|  | Yes | 1.27 | 1.12-1.44** | 1.56 | 1.38-1.76** |

*Note:* CI, confidence interval; OR, odds ratio. Values adjusted for gender, age, education level, marital status, living arrangement, monthly income, residence type, locality, national basic livelihood recipient status, smoking, drinking, high intensity exercise, moderate intensity exercise, walking, BMI, sleep duration, subjective stress level, depression, hypertension, diabetes, hyperlipidemia, arthritis, number of chronic illnesses, and subjective health status.

**p* < 0.05, ***p* < 0.001.
